# Characteristics of Inherited Metabolic Disorders Following Kidney Transplantation: A 13-Year Observational Study

**DOI:** 10.3390/medicina60111733

**Published:** 2024-10-22

**Authors:** Kirsty Dickson, Henry H. L. Wu, Reena Sharma, Karolina M. Stepien, Ana Jovanovic, Rajkumar Chinnadurai

**Affiliations:** 1 Faculty of Biology, Medicine and Health, The University of Manchester, Manchester M1 7HR, UK; kirsty.dickson@student.manchester.ac.uk; 2 Renal Research Laboratory, Kolling Institute of Medical Research, Royal North Shore Hospital, The University of Sydney, Sydney, NSW 2065, Australia; 3 Department of Adult Inherited Metabolic Diseases, Salford Care Organization, Northern Care Alliance NHS Foundation Trust, Salford M6 8HD, UK; reena.sharma@nca.nhs.uk (R.S.); karolina.stepien@nca.nhs.uk (K.M.S.); ana.jovanovic@nca.nhs.uk (A.J.); 4 Department of Renal Medicine, Salford Care Organization, Northern Care Alliance NHS Foundation Trust, Salford M6 8HD, UK

**Keywords:** inherited metabolic disorders, kidney transplantation, post-transplant care, clinical outcomes

## Abstract

*Background and Objectives:* Inherited metabolic disorders (IMDs), primarily cystinosis, Fabry disease, and methylmalonic acidemia (MMA), are genetic conditions that typically result in multi-organ disease manifestations. Kidney function progressively deteriorates in many cases, with patients eventually reaching end-stage kidney disease (ESKD) and requiring renal replacement therapy. Kidney transplantation has been deemed the optimal renal replacement therapy option to achieve long-term survival in patients with IMD. Whilst improved long-term survival is expected, the patterns of clinical evolution for IMD after transplantation remain largely unknown. *Methods:* Our group conducted a retrospective observational study that included 37 adult patients with IMD (11 with cystinosis, 20 with Fabry disease, and 6 with MMA). The study evaluated the clinical status and progression of these patients following kidney transplantation between January 2010 and December 2023. *Results:* This generally resulted in good graft outcomes for patients with IMD. Standard immunosuppression regimes included tacrolimus, mycophenolate mofetil, and prednisolone. The mean graft survival duration was noted to be 12 years in patients with cystinosis, 11 years in patients with Fabry disease, and 7 years in patients with MMA. Suboptimal outcomes were noted with grafts of cadaveric origin and poor adherence to the prescribed post-transplant immunosuppression regime. A greater extra-renal morbidity burden was associated with a reduced duration of graft function and increased mortality in patients with IMD. *Conclusions:* Our findings emphasise the need for a multi-disciplinary approach in the care of IMD patients following kidney transplantation.

## 1. Introduction

Inherited metabolic disorders (IMDs) are a heterogeneous group of genetic disorders. These conditions are typically monogenic and predominantly inherited in an autosomal recessive manner [1]. However, X-linked and autosomal dominant (AD) inheritance is also seen. Although the prevalence of an individual IMD is rare, IMDs have a high cumulative incidence, affecting 1/2500 live births [2]. There are currently 1450 well-characterised IMDs defined by the International Classification of Inherited Metabolic Disorders as any primary genetic condition in which the alteration of a biochemical pathway is intrinsic to specific biochemical, clinical, and/or pathophysiological features [3]. The majority of IMDs affect multiple organ systems, including the kidneys. The three most prevalent IMDs resulting in progressive kidney dysfunction and end-stage kidney disease (ESKD) include cystinosis (AR inheritance), Fabry disease (X-linked inheritance), and methylmalonic acidemia (MMA) (AR inheritance). Kidney transplantation is established as the optimal kidney replacement therapy option in terms of survival outcomes for patients with IMD and ESKD [4]. Whilst improved long-term survival is expected, the patterns of clinical evolution for IMD after transplantation remain largely unknown. These IMDs are multisystemic chronic progressive disorders, and patients have the additional burden of disease-specific therapies, which could be in the form of lifelong enzyme replacement therapy, substrate reduction therapy, or diet manipulation and restriction. These additional disease and treatment burdens could impact post-transplant renal outcomes and graft survival. Our study aimed to evaluate the characteristics and epidemiology of patients with these three IMDs after kidney transplantation and to identify any correlating factors that could influence graft survival.

## 2. Materials and Methods

### 2.1. Patient Selection

This single-centre observational study was conducted on adult patients with a confirmed biochemical and genetic diagnosis of one of three diseases—cystinosis, Fabry disease, or MMA—as per the guidelines of the International Classification of Inherited Metabolic Diseases workgroup [3]. Included patients received at least one kidney transplant for end-stage kidney disease secondary to one of the three IMDs and were followed up at the adult renal–metabolic medicine multi-speciality clinic at our centre, Northern Care Alliance NHS Foundation Trust (NCA), between January 2010 and December 2023. NCA is one of five major tertiary referral centres in England for adult IMD care, serving over seven million people. Patients were typically seen on a biannual basis at the renal–metabolic medicine multi-speciality clinic. This study was registered and approved by the Research and Innovation department at NCA (Reference: 24HIP20). As the study was retrospective and the data were pseudonymised, consent from individual participants was not warranted.

### 2.2. Data Collection

Data collected from electronic patient records included baseline demographics, transplant graft status, medication history, and co-morbidities. All available estimated glomerular filtration rate (eGFR) data were recorded as serial values between the study baseline and endpoint. The baseline date was taken as the first time that the patient was seen at the renal–metabolic medicine multi-speciality clinic following kidney transplantation (including patients who had been transitioned from paediatric services). Patients were followed up until endpoints that included death or loss to follow-up due to transfer to a different centre for care or the arbitrary endpoint date of 10 April 2024. Availability of data also varied according to the frequency of presentation to the clinic. The categories of data measurements varied according to the patient’s IMD. Nevertheless, they fell under the categories of patient characteristics, post-transplant biochemical values, co-morbidities, and outcomes. The disease-causing mutation was documented for all patients in the Fabry disease cohort. These mutations were compared against mutants listed on the Fabry disease database [5].

### 2.3. Statistical Analysis

When describing the findings from data analysis, continuous variables were expressed as the mean (standard deviation (SD)), and categorical variables were expressed as numbers (percentages). The annual rate of change in eGFR (i.e., delta eGFR) was calculated using the linear regression slope. Linear regression analysis included all available eGFR values from the study baseline until the endpoint date. Only patients with a minimum of three eGFR values recorded and/or one-year follow-up data were included in the analysis. eGFR was calculated using the Modification of Diet in Renal Disease (MDRD) equation. Analysis was conducted in SPSS version 26 (registered with the University of Manchester).

## 3. Results

The total cohort included 37 patients who were either diagnosed with cystinosis (*n* = 11), Fabry disease (*n* = 20), or MMA (*n* = 6).

### 3.1. Cystinosis

Of the total 13 patients diagnosed with cystinosis in the metabolic unit, 11 patients had received at least one kidney transplant. For these 11 patients (Table 1), the mean age at baseline presentation to the adult renal–metabolic medicine multi-speciality clinic was 24 years, with a predominance of females (64%) and individuals of South Asian ethnic origin (64%). At baseline, six patients (54%) had a kidney transplant, one patient (9%) was on dialysis, and four patients (36.4%) had chronic kidney disease (CKD) and were transplanted subsequently during follow-up. The mean age at transplant was 9 years (in paediatric services), and transplants were predominantly cadaveric (82%). Standard immunosuppression included tacrolimus, mycophenolate mofetil, and prednisolone. The mean eGFR at baseline was 50 mL/min/1.73 m^2^, and the mean urine protein–creatinine ratio (uPCR) was 38.5 mg/mmol. The mean white cell cystine level was noted to be 1.68 nmol half cystine/mg protein.

The mean graft survival was 12 years. Over a mean follow-up of 6.5 years, four patients had a failed primary graft, all of which were of cadaveric origin. The first patient’s primary graft lasted 7 years, and the transplant biopsy showed interstitial fibrosis and tubular atrophy (IFTA) likely due to chronic allograft nephropathy. The patient received a further cadaveric transplant, which was functional for 2 years, and IFTA was also apparent on the kidney biopsy. The patient then received a third cadaveric transplant in 2023, which remained functional at the end of the follow-up period. The second patient’s failed graft lasted 6 months, and there was evidence of chronic antibody-mediated rejection (cAMR) in addition to immunosuppressant non-compliance. The third patient’s failed graft demonstrated evidence of IFTA on kidney biopsy. However, this lasted 33 years, surpassing the upper limit of the expected lifespan for a cadaveric kidney transplant [6]. The patient received a second cadaveric transplant, which remained functional at the end of the follow-up period. The fourth patient’s failed graft lasted 11 years, and the patient was non-compliant with their immunosuppressant regimen. This patient also received a second cadaveric kidney transplant, which remained functional at the end of the follow-up period. Over a median follow of 4.6 years, the median (interquartile range) delta eGFR of the cystinosis cohort was −0.88 (−0.90 to −0.37) mL/min/1.73 m^2^/year.

The cohort exhibited other co-morbidities. These included issues with mood (*n* = 5), corneal cystine deposits leading to photophobia and reduced visual acuity (*n* = 4), recurrent episodes of urinary or gastrointestinal infection (*n* = 4), neurological involvement in the form of cognitive impairment (*n* = 3) and benign intracranial hypertension (*n* = 1), psychological involvement in the form of anxiety and depression (*n* = 1), poor mobilisation secondary to renal osteodystrophy (*n* = 3), diabetes mellitus following kidney transplantation (*n* = 2), hypothyroidism (*n* = 5), needing a percutaneous endoscopic gastrostomy (PEG tube) (*n* = 3), needing growth hormone (GH) supplementation (*n* = 2), and tremor (*n* = 2).

### 3.2. Fabry Disease

Of the total 674 patients with Fabry disease in the metabolic unit, 20 patients had received kidney transplantation. For these 20 patients (Table 2), the mean age at baseline presentation to the joint renal–metabolic medicine clinic was 48 years, with a predominance of males (95%) and those of white ethnicity (100%). At baseline, 7 people (35%) had a kidney transplant, 2 (10%) were on dialysis, and 11 (55%) were patients with CKD who were transplanted subsequently during follow-up. The mean age at transplant was 45 years, and the transplants were predominantly cadaveric (55%). Standard immunosuppression included tacrolimus, mycophenolate mofetil and prednisolone. The mean eGFR at baseline was 49 mL/min/1.73 m^2,^ with a mean uPCR of 53 mg/mmol. The delta eGFR was −0.37 (−0.75 to 0.46) mL/min/1.73 m^2^/year in the Fabry disease cohort over a median follow-up of 8.5 years. The mean alpha-galactosidase A level at clinic presentation was 0.60 nmol/mL/hour (SD 0.78), and the mean lysosomal Gb3 level was 49 nmol/L (SD 58).

The mean graft survival was 12 years. Over a mean follow-up of 6.8 years, five patients had a failed primary graft, all of which were of cadaveric origin. The first patient’s failed graft lasted 10 years and demonstrated IFTA. The second patient’s failed graft lasted 28 years and demonstrated IFTA in addition to cAMR. The third patient’s failed graft lasted 4 years and demonstrated evidence of IFTA and cAMR on kidney biopsy. The remaining two patients had grafts that functioned for 10 years and 4 years, respectively, and the reason for failure was not known. No patient with a failed primary kidney graft received further transplantation.

Enzyme replacement therapy (Replagal^®^) was the most common type of treatment prescribed (65%). Reviewing the cardiac status of individual patients, four people had cardiac arrhythmia (ventricular), with three receiving implantable cardiac defibrillators. Left ventricular hypertrophy (LVH) was noted to be reported in most patients’ echocardiograms (90%). Out of the eight patients who were deceased by the end of the follow-up period, 4 had a failed primary kidney graft. Of these patients, one patient died because of concurrent myocardial infarction and cerebrovascular accident. The cause of death was not recorded for the remaining patients; however, the predominant co-morbidities were cardiac and typically included end-stage heart failure.

All patients in the Fabry disease cohort had missense mutations, and the projected phenotypes were either severe or attenuated. Two patients with failed grafts had the R112C mutation, both of which were hemizygous. The R112C mutation was not observed in patients with functional grafts. This mutation is associated with a severe Fabry disease phenotype (the classic phenotype). One of the hemizygous patients with the R112C mutation was also found to have the R118C variant. Another patient with a failed graft was hemizygous for the I117S mutation (classic phenotype). This was also observed in two other patients with functional grafts. The remaining patients with failed grafts were hemizygous for L36W and I242F, neither of which neither had a known projected phenotype. Four patients with successful kidney grafts were hemizygous for the N215S mutation, associated with the variant or cardiac-predominant phenotypes.

### 3.3. Methylmalonic Acidemia

Of the total 16 patients diagnosed with MMA in the metabolic unit, 6 patients had received kidney transplantation. For these six patients (Table 3), the mean age at baseline clinic presentation was 20 years, with a predominance of males (83.3%) and with a 50:50 distribution of individuals of White and South Asian ethnicity. The cohort predominantly had MMA (83%), and only a single patient had combined MMA and homocystinuria phenotype. At baseline, five patients (83%) had a kidney transplant, with one patient (17%) on dialysis. The mean age at transplant was 15 years, which was predominantly cadaveric (66.6%). Standard immunosuppression included tacrolimus, mycophenolate mofetil, and prednisolone. The mean eGFR at baseline was 52 mL/min/1.73 m^2^. The mean MMA level at presentation to the clinic was 550 µmol/L (SD 220).

The mean graft survival was 6.7 years. Over a mean follow-up of 8.1 years, four patients had a failed primary graft. The first patient’s failed graft was cadaveric, lasted 1 year, and demonstrated evidence of cAMR. The patient received a second transplant of an unknown type, which remained functional at the end of the follow-up period. The second patient’s failed graft was from a live donor, lasted 12 years, and demonstrated evidence of IFTA. The patient did not receive further transplantation. The origin of the third patient’s failed graft was unknown; however, it lasted 13 years and demonstrated evidence of IFTA. The patient then received a second kidney transplant from a live donor, which remained functional at the end of the follow-up period. The fourth patient’s failed graft was cadaveric, lasted 6 years, and demonstrated evidence of both T-cell mediated rejection and IFTA. This patient did not receive further transplantation.

## 4. Discussion

Our study demonstrated associations in clinical characteristics between those with a failed primary graft and those in whom the graft remained functional at the end of the follow-up period. Established risk factors for poor graft prognosis in the wider population of kidney transplant recipients were also evident in our cohort. Such factors negatively affect the long-term outcome of kidney transplantation, leading to graft loss as a result of progressive kidney dysfunction. Delayed and progressive graft dysfunction are all poor prognostic indicators of graft outcome and are associated with unstable eGFR values and raised serum creatinine concentration [7].

The majority of patients in this study with a failed primary kidney graft, regardless of their IMD aetiology, were recipients of cadaveric transplants. Whilst the long-term prognosis for patients with ESKD is improved upon cadaveric transplant compared with dialysis, it is widely recognised that living donor grafts have increased survival compared with cadaveric grafts [8]. Furthermore, there is evidence to suggest that transplant recipients with marked extra-renal morbidity benefit particularly from living donor graft transplants [9].

Upon considering the limitations of our study, patients received transplants as early as 1980 and until 2023. Over this time period, surgical techniques have improved significantly, favouring better post-transplant outcomes [10]. The spectrum of immunosuppressant medications has become more effective and widely available [11], in addition to improved donor matching and post-transplant care [12]. Furthermore, there have been significant advances in the supportive management of IMDs, in particular for cystinosis [13]. The first disease-specific treatment for Fabry (Fabrazyme) was granted a marketing authorisation on 3 August 2001 in Europe. Consequently, the supportive management of these conditions has improved, hence likely resulting in reduced extra-renal morbidity. This is an important advancement, as extra-renal morbidity is a risk factor for poor post-transplant prognosis [14]. Therefore, any difference in kidney transplant outcomes between patients over this time period may partially account for by these factors rather than patient-specific, baseline characteristics.

Non-adherence to immunosuppressants has been established as a significant risk factor for poor outcomes in kidney transplantation [15]. Our study suggested that immunosuppressant non-compliance was the likely underlying cause of transplant failure in two patients with cystinosis, demonstrating the importance of regular patient review and meticulous exploration of non-adherence to post-transplant immunosuppression regimens. Both immunological and non-immunological factors of graft failure impact long-term outcomes related to the primary IMD diagnosis, for example, childhood growth and the risk of bone disease in cystinosis.

Regarding cystinosis, there are limited previous data on the relationship between extra-renal disease burden and kidney transplant outcomes in patients with cystinosis. Despite four patients experiencing primary kidney graft failure, there was a period of reasonable graft function. The results from this study suggest that cystinosis patients with a higher baseline eGFR value had a longer post-transplant survival. Spicer et al. [16] confirmed that higher eGFR values may reflect cysteamine use and hence better systemic disease management. Higher baseline eGFR values may also reflect pre-emptive transplantation in paediatric patients, reflecting a recent change in the indication for transplant, likely pertinent to this study. Emma et al. [17] report that despite successful kidney transplantation, many patients do not survive beyond the age of 30 years if they have not been treated with cysteamine, which is known to reduce the extra-renal burden of cystinosis. Whilst the extent of any such associations remains unknown, the results of this study do indicate that patients with greater extra-renal morbidity at baseline, such as those requiring PEG tube or GH replacement and those with vitamin D deficiency, were more likely to have failed grafts.

Findings from the Fabry disease cohort suggested that increased severity of pre-existing extra-renal disease was negatively associated with graft function. Cardiovascular morbidity has been established as a poor prognostic indicator in the general population of kidney transplant recipients [18]. Capelli et al. [14] reported that long-term graft survival is reduced by cardiovascular morbidity and cardiovascular-associated mortality in patients with Fabry disease. In this study, Fabry disease patients with a failed graft had disease-causing mutations associated exclusively with the classic Fabry disease phenotype. These mutations are associated with a higher cardiovascular burden than attenuated phenotypes. Furthermore, four of five patients with a failed graft were deceased at the end of the study period, representing half of the deceased patients. Most of these patients had end-stage cardiovascular disease. However, whilst all patients with a failed graft in the study demonstrated evidence of LVH at baseline, no patients had arrhythmia or an implantable cardioverter defibrillator (ICD). Therefore, whilst cardiovascular morbidity adversely affects post-transplant outcomes, it may not always be evident at baseline.

The results yielded from the MMA cohort were inconclusive. This cohort was small, the data set was incomplete, and those with a failed primary graft represented much of the patient cohort. Wider evidence concerning factors that affect transplant outcomes in patients with MMA is lacking, likely due to the rare nature of the disease and heterogeneity within the condition itself. Larger cohorts and longer follow-up periods are necessary to determine whether any demographic factors influence transplant outcomes and the extent of any such variation.

The relatively stable nature of delta eGFR in the linear regression slopes suggests that kidney transplantation produced better outcomes in patients with cystinosis and Fabry disease compared with those not receiving transplantation, in which eGFR would likely decrease until minimal eGFR or complete loss of kidney function. Despite variations in the relative efficiency of the graft organ, these results support the premise that kidney transplantation leads to good outcomes in patients with IMD [4]. Overall, this study was concordant with the wider consensus that kidney transplantation was safe and typically provided optimal outcomes in IMD patients with ESKD [19].

There are evident limitations of our study. The key limitation is the study design’s retrospective nature and variable follow-up period within each cohort. This meant that graft survival could not be recognised. Furthermore, it was not possible to recognise patients due to their variable kidney status upon initial presentation. Whilst the indication for kidney transplantation was assumed to be ESKD, this has not been fully explicit in clinical documentation. This means that some patients may have received pre-emptive transplantation without dialysis, which was associated with improved graft outcomes [20]. The rare nature of these conditions led to small cohort groups, which made it difficult to determine whether any difference in baseline characteristics was incidental since robust statistical analysis was not feasible. The small cohorts also limited variation with regard to sex and ethnicity. Hence, it was not possible to discern the effect of such variables. This was compounded by incomplete datasets, and important clinical parameters, such as human leukocyte antigen mismatch status and uPCR, were not well-documented.

Overall, patients with IMD only represent a very small proportion of all kidney transplant recipients. Therefore, it would be difficult to compare the relative impact of demographic factors specific to IMD on graft failure and compare these to known risk factors within the general population. Specifically, whilst extra-renal morbidity is an established risk factor for poor kidney transplant outcomes, the relative contribution of extra-renal morbidity towards graft failure remains unknown due to the rare and variable nature of IMDs, and the mechanism by which extra-renal morbidity affects kidney transplant outcomes are not yet fully understood. Compliance with anti-graft rejection medications may have been impacted in patients with cystinosis who have a much higher therapy burden. Finally, some studies suggested that the event of kidney transplantation itself is an instigator to unmask the extra-renal manifestations of IMDs, such as the case of cystinosis, and it should not be considered as a parameter to predict graft outcomes [16].

## 5. Conclusions

This study recognises the importance of kidney transplantation in managing patients with IMD. The patient cohorts with cystinosis and Fabry disease demonstrated good overall graft outcomes. A greater extra-renal morbidity burden was demonstrated to be associated with a reduced duration of graft function and increased mortality in patients with IMD. Ultimately, our findings emphasise the advantages of a multi-disciplinary approach in the care of patients with IMD following kidney transplantation, with concurrent and integrated management for both renal and extra-renal disease manifestations to promote positive clinical outcomes. Collaboration across specialities will be key in addressing the complex needs of patients with IMD after transplantation, ensuring that care is holistic and covers both the metabolic and immunological aspects. The emotional and psychological impact of disease and treatment received also needs consideration, and psychosocial support in this relatively young cohort of patients with IMD is needed. This could improve patient compliance with medications. Furthermore, investigating gene therapy and CRISPR-based interventions alongside transplantation might represent a promising area of future research. Newborn screening and next-generation gene testing are expanding. These measures could allow patients to be diagnosed and access treatments much earlier, potentially avoiding end-organ damage.

## Figures and Tables

**Table 1:** Baseline characteristics and outcomes in patients with cystinosis.

Characteristic (*n*=11)		Mean [SD] and Number (%)
Age at diagnosis, years		8.5 [7.2]
Age at clinic presentation, years		24 [7.57]
Sex	Female	7 (63.6%)
Ethnicity	British	4 (36.4%)
	South Asian	7 (63.6%)
BMI, kg/m^2^		24.4 [2.9]
Renal status at presentation	Transplanted	6 (54.5%)
	Dialysis	1 (9.0%)
	CKD	4 (36.4%)
Age at transplant, years		9 [6.26]
Type of transplant	Cadaveric	9 (82%)
	Live	2 (18%)
Immunosuppression at presentation	Tacrolimus	10 (90.9%)
	MMF	7 (63.6%)
	Prednisolone	9 (81.8%)
Post-transplant biochemical values on presentation		
eGFR, mL/min/1.73 m^2^		50 [34.1]
uPCR, mg/mmol		38.5 [23.0]
Baseline WCC, nmol 1/2 cystine/mg protein		1.68 [1.66]
Baseline Vitamin D, nmol/L		43.1 [28.4]
Co-morbidities		
Baseline Thyroid Status	Normal	6 (54.5%)
	Hypothyroid	5 (45.5%)
PEG		3 (27.3%)
GH Supplementation		2 (18.2%)
Outcomes		
Follow-up, years		6.5 [3.9]
Alive (at end of follow-up)		11 (100%)
Graft function status (by end of follow-up)	Functional	7 (63.6%)
	Failed	4 (36.4%)
Graft survival duration, years	Entire cohort	11.8 [10.9]
	Functional	11.2 [9.9]
	Failed	12.8 [14.1]

BMI: Body mass index; CKD: Chronic kidney disease; eGFR: Estimated glomerular filtration rate; GH: Growth hormone; MMF: Mycophenolate mofetil; PEG: Percutaneous endoscopic gastrostomy; SD: Standard deviation; uPCR: Urine protein–creatinine ratio; WCC: White cell cystine; Continuous variables are expressed as means (standard deviation), and categorical variables are expressed as numbers (percentages).

**Table 2:** Baseline characteristics and outcomes in patients with Fabry disease.

Characteristic (*n* = 20)		Mean [SD] and Number (%)
Age at clinic presentation, years		48 [11.02]
Sex	Male	19 (95%)
	Female	1 (5%)
Ethnicity	White	20 (100%)
Body mass index, kg/m^2^		25.4 [4.27]
Renal status at presentation	Transplant	7 (35%)
	Dialysis	2 (10%)
	CKD	11 (55%)
Age at transplant, years		45 [14]
Type of transplant	Cadaveric	11 (55%)
	Live	9 (45%)
Immunosuppression at presentation	Tacrolimus	16 (80%)
	MMF	13 (65%)
	Prednisolone	16 (80%)
Type of mutation	N215S	4 (20%)
	I117S	3 (15%)
	R112C	2 (10%)
	Other	11 (55%)
Post-transplant biochemical values on presentation		
eGFR, mL/min/1.73 m^2^		49 [22.7]
uPCR		53 [77.8]
Co-morbidities		
Cardiac Arrhythmia		4 (20%)
ICD		7 (35%)
LVH		18 (90%)
ERT	Replagal	13 (65%)
	Fabrazyme	7 (35%)
Outcomes		
Follow-up, years		6.8 [3.9]
Alive (at end of follow-up)		12 (60)
Deceased (at end of follow-up)		8 (40)
	Age at death, years	55 [10.7]
Graft function status (by end of follow-up)	Functional	15 (75)
	Failed	5 (25)
Graft survival duration, years	Entire cohort	11.0 [9.2]
	Functional	10.1 [9.1]
	Failed	14.6 [9.7]

CKD: Chronic kidney disease; eGFR: Estimated glomerular filtration rate; ERT: Enzyme replacement therapy; MMF: Mycophenolate mofetil; ICD: Implantable cardioverter defibrillator; LVH: Left ventricular hypertrophy; SD: Standard deviation; uPCR: Urine protein–creatinine ratio; Continuous variables are expressed as means (standard deviation), and categorical variables are expressed as numbers (percentages).

**Table 3:** Baseline characteristics and outcomes in patients with MMA.

Characteristic		Mean [SD] and Number (%)
Age at clinic presentation, years		20 [3.9]
Sex	Male	5 (83.3)
	Female	1 (16.7)
Ethnicity	White	3 (50)
	South Asian	3 (50)
MMA type	Isolated	1 (17)
	Combined	5 (83)
Body mass index, kg/m^2^		24.6 [4.7]
Renal status at presentation	Transplant	5 (83.3)
	Dialysis	1 (16.7)
	CKD	0 (0)
Age at transplant		15 [9]
Type of transplant	Cadaveric	4 (66.6)
	Live	1 (16.6)
	Unknown	1 (16.6)
Immunosuppression at presentation	Tacrolimus	6 (100)
	MMF	6 (100)
	Prednisolone	3 (50)
Post-transplant biochemical values at presentation		
eGFR, mL/min/1.73 m^2^		52 [45.1]
Co-morbidities		
PEG		5 (83.3)
Outcomes		
Follow-up, years		8.1 [4.2]
Alive (at end of follow-up)		6 (100)
Graft function status (by end of follow-up)	Functional	2 (33.3)
	Failed	4 (66.7)
Graft survival duration, years	Entire cohort	7.1 [4.9]
	Functional	4.8 [4.4]
	Failed	8.2 [5.3]

CKD: Chronic kidney disease; eGFR: Estimated glomerular filtration rate; MMA: Methylmalonic acidaemia; MMF: Mycophenolate mofetil; PEG: Percutaneous endoscopic gastrostomy; SD: Standard deviation; Continuous variables expressed are as means (standard deviation), and categorical variables are expressed as numbers (percentages).

## Data Availability

Data are available upon reasonable request to the authors.
